# RNA Activation in Plants: Current Evidence and Molecular Insights

**DOI:** 10.3390/plants15192956

**Published:** 2026-09-28

**Authors:** Alexandra S. Dubrovina, Konstantin V. Kiselev

**Affiliations:** Laboratory of Biotechnology, Federal Scientific Center of the East Asia Terrestrial Biodiversity, Far Eastern Branch of the Russian Academy of Sciences, 690022 Vladivostok, Russia; kiselev@biosoil.ru

**Keywords:** RNA activation, small activating RNAs (saRNAs), double-stranded RNA (dsRNA), gene expression, transcriptional activation, exogenous RNA, small RNA, DNA methylation, RNA interference (RNAi), small interfering RNAs (siRNAs)

## Abstract

The current scientific literature presents increasing evidence for the existence of the RNA activation (RNAa) phenomenon in eukaryotes. RNAa is a molecular mechanism by which gene expression is activated through small activating RNAs (saRNAs) and microRNAs (miRNAs). The RNAa pathway shares many similarities with RNA interference (RNAi), yet it represents a distinct regulatory process. While RNAi is a relatively well-characterized process involving Dicer-like endonucleases that process double-stranded RNAs (dsRNAs) into small interfering RNAs (siRNAs), ultimately leading to gene silencing, the molecular mechanisms underlying RNAa remain poorly understood. This review summarizes and discusses the current knowledge of RNA-mediated activation of gene expression in plants, highlighting this emerging research area beyond gene silencing. While research on RNAa in plants remains limited, available data support the existence of RNA-triggered gene activation and RdDM-associated transcriptional derepression at specific plant loci. Whether these phenomena constitute a unified RNAa pathway analogous to that described in animals remains to be determined. A broader understanding of RNAa-related processes is essential for advancing plant science and developing innovative strategies in plant molecular biology and biotechnology.

## 1. Introduction

A number of intriguing reports have appeared in the scientific literature, demonstrating the existence of a gene activation mechanism mediated by small RNAs, termed the RNA activation (RNAa) phenomenon in eukaryotic cells, including humans, monkeys, mice, nematodes, and some insects. The term RNAa was coined in 2006 [1] as a gene regulation mechanism in which small double-stranded RNAs (dsRNAs), including small activating RNAs (saRNAs) or activating microRNAs (miRNAs), upregulate gene expression while interacting with protein co-factors and chromatin remodelers to promote RNA polymerase activity and gene expression [2,3,4,5,6]. In its original and most widely accepted sense, RNAa refers to a small RNA-guided, Argonaute (AGO)-dependent phenomenon in which small promoter-targeted dsRNAs, termed saRNAs, induce transcriptional activation of target genes via epigenetic remodeling [1]. This definition encompasses two key features: (1) the effector molecule is an RNA, and (2) the mechanism is AGO-dependent and targets gene regulatory sequences to modulate transcription. For mammals, the effector molecule is a short dsRNA (i.e., saRNA) or a miRNA acting in a saRNA-like manner. For other organisms such as insects and plants, it can also be a long dsRNA.

The term RNAa was introduced in contrast to the phenomenon of RNA interference (RNAi), where gene expression is reduced with the assistance of small interfering RNAs (siRNAs) and miRNAs [7,8,9]. In RNAa, small RNAs promote gene expression via transcriptional gene activation, whereas in RNAi, they mediate mRNA degradation and translational repression. In contrast to RNAa, the process of RNAi is much better studied. During RNAi, dsRNAs are processed by specialized enzymes, Dicer in animals or Dicer-like (DCL) in plants, into siRNAs duplexes. One strand of each duplex is then selected and loaded into the RNA-induced silencing complex (RISC), which uses it to guide cleavage of complementary mRNA targets, thereby reducing expression of the corresponding genes at the post-transcriptional level [7,10]. miRNAs, in turn, are genome-encoded small RNAs of 20–24 nt that are generally known to regulate gene expression at the post-transcriptional level, primarily in the cell cytoplasm, by guiding mRNA degradation or translational repression [11,12]. RNAi was originally defined as a dsRNA-induced, siRNA-mediated silencing pathway. The miRNA pathway, in contrast, is mechanistically related, but historically distinct, sharing the core components of the silencing machinery such as Dicer, AGO, and RISC.

In 2006, Li and co-authors discovered that targeting promoters of three human genes with synthetic small dsRNAs can stably induce target gene expression at the transcriptional level in human cultured cells [1]. This new phenomenon of gene regulation was designated as RNAa. The saRNAs are structurally similar to siRNAs, having a 19-nucleotide (nt) duplex with 2-nucleotide protrusions, and have been named saRNAs. This discovery was soon confirmed by Janowski et al. on the progesterone receptor gene in human cancer cell lines and has been shown to act through sequence-specific interactions at the PR promoter [13]. Since then, a number of publications on mammals and insects have appeared, which can be found in review papers [3,4,5,6,14], where the phenomenon of RNAa has been confirmed in cells of various mammals, including human cells, African green monkeys, chimpanzees, mice, rats, and insects.

RNA activation process is known to be mediated not only by saRNAs but also by activating miRNAs. Traditionally, miRNAs are recognized for their role in gene silencing, where they promote mRNA degradation or inhibit translation during the RNAi process [11,12]. It should be noted that saRNAs in the context of RNAa are usually considered as short dsRNA molecules that are artificially introduced during experiments or enter cells from external natural sources. In contrast, activating miRNAs are of endogenous origin and are processed through transient double-stranded intermediates: primary miRNAs (pri-miRNAs) are processed in the nucleus into shorter precursors (pre-miRNAs), which are further cleaved by DCL1 in the nucleus (in plants) or Dicer in the cytoplasm (in animals) into short miRNA duplexes, from which the single-stranded mature miRNA guide strand is subsequently selected and loaded into an AGO complex [4,8,11,12]. Existing data show that miRNAs can also directly or indirectly up-regulate gene transcription [4,15,16,17] and even translation [18,19]. Increasing evidence demonstrates that miRNAs can be found in the nucleus, where they participate in transcriptional gene regulation by either activating or silencing gene expression [20,21,22,23]. Such nuclear miRNAs with gene-activating functions have been named nuclear-activating miRNAs or namiRNAs [24].

While research on RNAa in plants is currently limited, the available evidence points to this phenomenon and warrants closer analysis to guide future progress in this field. In this review, we first provide an overview of the current understanding of RNAa mechanisms and their roles in mammals, and then focus on the emerging evidence for nucleic acid-mediated gene activation in plants to show recent advances and remaining knowledge gaps.

## 2. RNA Activation Mechanisms: What Is Known for Eukaryotes

Currently, the molecular mechanisms of RNA-mediated gene activation have been investigated primarily in mammals, predominantly in human cancer cell lines, while mechanistic evidence for plants and other organisms remains limited. The initial scholarly publications documenting the activation of gene expression via small RNAs were published approximately two decades ago. In 2004, Kuwabara et al. [25] discovered that small noncoding dsRNAs of about 20 bp play a critical role in mediating neuronal differentiation by modulating the expression of neuron-specific genes. Two years later, Li and colleagues [1] exogenously applied synthetic small dsRNAs of 21 bp complementary to the promoter regions of several human genes (E-cadherin, p21, and VEGF) to human cancer and embryonic cell lines. They discovered that these dsRNAs induced a sequence-specific and sustained increase in the levels of targeted mRNAs in the human cancer cell lines. This phenomenon was termed RNAa, and the dsRNAs involved were designated as saRNAs in subsequent studies.

In 2007, Janowski et al. [13] demonstrated the ability of small dsRNAs (19–21 bp) targeting the promoter of the progesterone receptor (PR) gene to enhance its mRNA and protein levels in breast cancer cell lines, corroborating the data of Li et al. [1]. Subsequent studies confirmed the results and expanded the understanding of RNAa by demonstrating that synthetic short dsRNAs complementary to sequences beyond the 3′ UTR of the PR mRNA trigger RNAa-based gene activation in human cancer cell lines [26]. In this paper, Yue et al. [26] reported that small dsRNA-mediated activation preferentially occurs in cells with low basal PR gene expression, whereas gene silencing is more commonly observed in cells with high levels of PR gene expression. These findings suggest that small RNA-mediated regulation can result in either gene activation or gene silencing, depending on the cellular context and transcriptional state of the target gene. Subsequently, numerous studies have demonstrated, as reviewed in [3,4,5,6,14], that small dsRNAs can activate gene transcription, indicating that the influence of these small RNAs extends beyond the traditional view of them as primarily negative regulators and includes positive regulation of gene expression. These small RNA molecules have been referred to in different publications as antigene RNAs (agRNAs) [13,26], small modulatory RNAs (smRNAs) [25], or small activating RNAs (saRNAs) [27]. At present, the most widely accepted and established terminology for these entities is saRNAs [3,5,14].

While the RNAa phenomenon was initially identified through the exogenous application of synthetic small dsRNAs, subsequent reports have documented the capacity of endogenous miRNAs to upregulate mRNA transcription in mammals and some other non-plant systems, as reviewed in [4,21,22,23,28], and even activate protein translation [18,19]. For instance, Place et al. [16], which is one of the first studies in this field, showed that the transfection of miR-373 and its precursor into prostate cancer cells induces the expression of *E-cadherin* and cold-shock domain-containing protein C2 (*CSDC2*) genes, which possess a putative miR-373 target site within their promoters. Turner et al. [17] expanded this concept by showing that RNAa is a physiological mechanism, not just an effect of artificial miRNA transfection. Using the nematode model *Caenorhabditis elegans*, the authors demonstrated that the miRNA lin-4 activates its own transcription (positive feedback, autoregulation) through RNAa, ensuring lin-4 expression maintenance during development. These results provided early evidence that miRNA-mediated transcriptional activation functions in vivo as a natural regulatory mechanism. Xiao et al. [15] further advanced the understanding of the RNAa concept by using the human miRNA miR-24-1 to demonstrate that miRNAs do not function exclusively at promoters. It has been shown that they can localize to the nucleus, bind tightly to enhancers (cis-acting DNA elements that boost transcription), alter local epigenetic marks, initiate chromatin remodeling in the enhancer region, and stimulate expression of enhancer RNA to powerfully activate gene transcription of their neighboring genes [15]. The miR-24-1 miRNA increased the enrichment of the histone acetyltransferase p300 and RNAPII at the enhancer, which physically induced the expression of the target genes. These and other available observations demonstrate that activating miRNAs can be involved in the regulation of various biochemical pathways and diseases, contributing to the upregulation of gene transcription within the nucleus [21,22,23,28]. Overall, accumulating evidence from animal and plant models indicates that a subset of mature nuclear-localized miRNAs, termed namiRNAs, have been implicated in modulating transcription by either promoting transcriptional silencing or transcriptional activation through interactions with chromatin or components of the transcriptional machinery [21,22,24,29]. Nuclear localization alone, however, is not sufficient evidence of a transcription-regulatory role.

Further investigations revealed that the mechanism by which small activating dsRNAs exert their effects is a complex process involving a series of molecular interactions (Figure 1). saRNAs have been shown to interact with protein co-factors involved in RNA polymerase II (RNAPII)-dependent transcription [30] and to induce the formation of nucleosome-depleted chromatin regions at target loci [31]. Upon cellular uptake, saRNAs associate in the cytoplasm with dsRNA-binding proteins, such as several heterogeneous nuclear ribonucleoproteins (hnRNPs) A1, A2/B1 and C1/C2 [32], and then are preferentially loaded into the AGO2 [33,34] or AGO1 proteins [35] (Figure 1). AGO1 and AGO2 proteins are members of the Argonaute family known for their role in RNAi and gene silencing [36]. The variability in AGO protein interactions suggests that different saRNAs can modulate gene expression through distinct pathways. Then, according to the current working model (Figure 1), the complex comprising guide saRNA, hnRNPs, and AGO is relocated to the nucleus, where it directly interacts with DNA, thereby promoting the formation of an RNA-induced transcriptional activation (RITA) complex as shown in Portnoy et al. [30].

Currently, it is a debated question whether DNA or RNA is a target for saRNAs. A number of studies show that saRNAs/miRNA bind to the genomic target sites (1) within or near the promoter [37,38], (2) regions downstream of the 3′ UTR [26], or (3) interact with enhancer-associated chromatin [15]. A number of studies demonstrated binding of saRNA/miRNA to the noncoding RNA, frequently termed promoter-associated RNA (paRNA), that overlaps with the gene targets within promoter or 3′ terminal gene regions [34,39,40]. Finally, an RNA:DNA triplex model was proposed, reporting that purine or pyrimidine-rich miRNAs can establish triple-helical architectures within the major groove of duplex DNA [41]. This occurs via Hoogsteen or reverse Hoogsteen base-pairing, a mechanism supported by conclusive evidence presented by Paugh et al. [41]. Thus, the RNA-AGO complex is proposed to engage genomic promoters or other genomic regions via three alternative interaction modes, specifically through RNA:RNA, RNA:DNA, or triple-helical RNA-DNA configurations [42].

For synthetic saRNAs, assembly of the RITA complex involves several crucial components: RNA helicase A (RHA), Cln three requiring 9 (CTR9), and DEAD-box helicase 5 (DDX5) [30,43]. CTR9 is a part of the Polymerase Associated Factor 1 Complex PAF1C [44]. This complex is crucial for controlling the initiation and continuation of transcription processes. It interacts with histone-modifying enzymes and RNAPII, thereby influencing the transcriptional machinery. Beyond its primary role as an ATP-dependent helicase that unwinds RNA and DNA, RHA also recruits chromatin remodelers and other components that are necessary for constructing the transcriptional activation complex. As a member of the DEAD-box protein family, DDX5, also known as ATP-dependent RNA helicase 5, participates in many cellular processes, e.g., initiation of translation or splicing, that require modifications to RNA secondary structures. Finally, the collective action of the RITA complex facilitates the recruitment of RNAPII and ensures transcription initiation and productive elongation [30,43].

Currently, much remains unknown about the structural characteristics of saRNAs and activating miRNAs, which is very important for understanding the rules governing their design for successful experiments. However, existing studies provide the following information about the general features of activating small RNAs. First, the antisense strand of active saRNA was effectively integrated into AGO2, while the sense strand did not exhibit such integration [1,38]. Furthermore, it has been shown that the saRNA-mediated gene activation depends on the complementarity between the 5′ end of the antisense chain and sequences inside or near the promoter region of the target gene [1,38]. It has been demonstrated that saRNAs operate through a localized mechanism by binding to target genomic DNA strongly depending on the seed region, similar to the miRNA-mediated target recognition process [38]. Taken together, the analysis of the available literature data [1,13,16,38,45,46,47,48,49,50,51,52,53,54,55,56,57,58,59] summarized in Table S1 revealed that the length of activating saRNAs and miRNAs was remarkably conserved at 19–23 nt across virtually every system and every target-region type (promoter, enhancer, 3′-UTR, coding sequence). Also, most used saRNA/miRNA mimic were unmodified in the analyzed studies (Table S1). There are several exceptions, e.g., (1) the TATA-box-optimized interleukin IL-2 saRNA (2′-O-Me at the 3′ antisense terminus and stability/specificity optimization) [45] or (2) the clinical-stage transcription factor CCATT/enhancer binding protein alpha CEBPA saRNAs (different patterns of 2′-O-Me modifications) [49,50]. The data also show that the mammalian RNAa studies primarily focus on 19–21 nt saRNAs, specifically because long dsRNA (roughly >30 bp) in mammals is a potent trigger of the innate antiviral response, which could confound the results of studies related to transcription activation due to a general stress or immune response. However, it should be noted that some available studies on non-mammalian systems such as insects [56,57] and plants [60,61] analyzed long dsRNAs as inducers of gene activation (Table S1).

Current evidence reveals that miRNA-mediated RNAa involves miRNAs that guide protein complexes to promoter-paused RNAPII, promoting its release into productive elongation. Ohno et al. [40] found that activating miRNAs recognize paRNAs and recruit AGO-containing protein complexes to gene promoters, which eventually leads to chromatin remodeling and enhanced transcription. They demonstrated that endogenous miR-34a in a human lung cancer cell line activates a tumor suppressor gene, Zinc Finger MYND-Type Containing 10 (*ZMYND10*), by targeting such paRNAs and forming an AGO2-TNRC6 protein complex, which recruits the RNA helicase DDX21 [40]. This enables CDK9 to phosphorylate RNAPII, triggering its release from the paused state into productive transcription elongation. However, the universality of this mechanism and its physiological significance remain to be established. In a different paper, Luo et al. [62] showed that miR-150 activated perilipin-2 (*PLIN2*) transcription through direct interaction with paRNA transcripts overlapping the *PLIN2* promoter and facilitating the recruitment of DNA helicase DHX9 and RNAPII. This eventually enhanced hepatic lipid accumulation during fatty liver development.

As research progressed, it became evident that the phenomenon of RNAa is not limited to human cells but is present across a wide range of eukaryotic organisms. RNAa has been documented in organisms ranging from the nematode *C. elegans* [17] to human cells and cells of other mammals, including monkeys [63], chimpanzees [63], mice [52,64], rats [65], and domestic sheep [66]. Conclusive evidence for the phenomenon of RNAa has also been published for mosquitoes, *Aedes aegypti* [56], and ticks, *Haemaphysalis longicornis* [57]. Such a wide distribution of RNAa indicates the ubiquitous nature and evolutionary importance of this phenomenon. Although a relatively recent discovery, RNAa has established itself as a promising approach for modulating gene expression in oncology research or in treating other diseases such as metabolic disorders or Alzheimer disease [5,67]. Its principle of targeted transcriptional activation is important for therapeutic applications, e.g., for gain-of-function approaches involving tumor suppressor genes.

Recent research has challenged the conventional view of small RNAs as gene silencers and provided evidence of the dual nature of the action of miRNAs on gene expression. However, the existing literature lacks a unified model to explain how RNAa achieves its effects. Further investigation is essential to expand our understanding of the molecular mechanisms and functions of the RNAa phenomenon.

## 3. Plant RNAa-Related Mechanisms

### 3.1. Plant Gene Activation by RNA-Directed DNA Methylation (RdDM)

Several scientific investigations have provided evidence supporting the existence of RNAa-related processes in plant systems [60,61,68,69,70,71,72,73,74], as summarized in Table 1. However, it should be emphasized that empirical evidence for this phenomenon in plants is currently limited. An essential reason for considering RNAa in plants is the evolutionary conservation of key RITA components that have functional homologs in plant cells [75,76]. Specifically, the PAF1C complex is present in plants: in *Arabidopsis thaliana*, it comprises six subunits as in other multicellular organisms [76]. The subunit corresponding to CTR9 in PAF1C is the ELF8/VIP6 protein in *A. thaliana*. The other subunits are encoded by the *ELF7*, *VIP3*, *VIP4*, *VIP5*, and *CDC73* genes in *A. thaliana*. This complex is conserved across eukaryotes and positively regulates transcriptional elongation by RNAPII in plants, as in other organisms. It should be noted that the conservation of some of these complex components in plants provides a molecular basis worth investigating, but it does not currently demonstrate the presence of a RITA-like complex in plant cells. Whether plant PAF1C subunits such as ELF8/VIP6 associate with AGO-small RNA complexes in a manner analogous to the RITA complex in mammals remains an open question for future experimental work.

Shibuya et al. [60] and Kapoor et al. [77] demonstrated that the introduction of a part of the *pMADS3* genomic sequence into Petunia plants leads to the ectopic activation of the *pMADS3* gene, an unprecedented type of transgene-induced, homology-dependent modulation of an endogenous gene (Table 1). This resulted in *pMADS3* expression in atypical plant tissues such as petals, sepals, and leaves and was accompanied by homeotic conversion of the floral organs and altered leaf morphology similar to that of an Arabidopsis curly leaf mutant. The Petunia *pMADS3* is a crucial class C MADS-box transcription factor gene, a homolog of the Arabidopsis *AGAMOUS* gene, and is responsible for the specification of stamens and carpels as well as for floral meristem maintenance. It is expressed in Petunia stamens and carpels during flower development. An important and surprising finding was that small dsRNAs derived from the introduced sequences triggered the DNA cytosine methylation process, ultimately activating the *pMADS3* gene [60]. It has been shown that this activation was linked to the methylation of specific CG sites within the putative negative cis-regulatory element of *pMADS3* intron 2. When DNA methylation takes place in a negative cis-element rather than in a positive cis-element, it can disrupt the binding of the corresponding transcriptional repressor to the cis-element, thereby resulting in transcriptional derepression as proposed in this study [60]. Notably, no alterations in histone modifications were observed [60], which indicates a unique mechanism of this activation in plants achieved through RdDM [78], while in mammals the mechanism is associated with histone modification [5]. This cytosine methylation was heritably maintained even without the original RNA trigger, leading to *pMADS3* transcriptional activation [60]. To the best of our knowledge, this observation first revealed a novel regulatory mechanism that upregulates gene expression in plants, contrasting with the widely known RNAi-based gene silencing effects.

DNA cytosine methylation, especially promoter methylation, is generally known to repress transcription, but in some studies (Table 1) it has been reported to activate gene expression in both plants and animals [73,74,79,80]. DNA methylation regulates gene expression by attracting specific proteins that control transcription. It hinders or, in turn, promotes the binding of transcription factors to DNA. The DNA methylation-mediated gene activation is less understood than its role in transcriptional silencing, which is well-established. For *A. thaliana*, Harris et al. [73] discovered that two SU(VAR)3-9 homologs (SUVH) proteins specifically bind to methylated DNA and recruit the DNAJ domain-containing (SDJ) proteins to activate the transcription of genes that are already mildly transcribed. This effectively neutralizes the inhibitory effects of transposon insertion close to these genes. In support of the positive role of DNA methylation, Zhao et al. [74] found that the components of the SUVH-SDJ complex are frequently found at methylated promoters targeted by RdDM and are essential for the expression of the corresponding genes. The SUVH-SDJ complex exhibited transcription-activating function, and this activation was crucial for plant growth and development. It should be noted that RdDM-linked “reader” complexes that activate transcription are downstream of RdDM, and the activation trigger itself is therefore not RNA, but the binding of reader proteins to already methylated DNA.

Collectively, these data suggest that RdDM [78], a pathway predominantly associated with transcriptional silencing, may also function as a mechanism for RNA-mediated gene activation in plants, leading to stable epigenetic states.

### 3.2. Exogenous dsRNA: From RNA Interference to Potential RNA Activation

The current research demonstrated that long dsRNA precursors and small dsRNAs can be applied externally to plant surfaces to induce RNAi-mediated gene silencing within plant cells or in invading plant pathogens [81,82,83,84,85]. This method is a novel approach in plant biotechnology and agriculture, frequently termed spray-induced gene silencing (SIGS) or exogenously induced RNAi (exo-RNAi) [81,82,83,84,85]. Exogenously added plant-specific or pathogen-specific dsRNAs, siRNAs, and miRNAs are known to be absorbed by plant tissues, penetrate plant cells, and are subsequently transported via the plant vascular system, resulting in the downregulation of the gene targets [85,86,87,88,89,90].

It appears that such an approach can be used in plants to study the roles of dsRNAs and small RNAs as mediators in both RNAa and RNAi. In one of our recent studies, we treated the leaves of tomato, *Solanum lycopersicum*, exogenously with synthetic dsRNAs designed to target *SlTRY*, a gene encoding an R3-type MYB transcriptional repressor of anthocyanin biosynthesis [61,91] (Table 1). Unexpectedly, dsRNA complementary to the intronic region of the *SlTRY* gene considerably increased the expression of the *SlTRY* gene after foliar *SlTRY*-dsRNA spraying under standard cultivation conditions. On the contrary, dsRNA targeting the coding region of *SlTRY* reduced its expression [61,91]. This dual outcome indicates that plant cells can interpret exogenous dsRNAs in a location-specific manner, potentially activating or repressing gene expression depending on the targeted genomic region. These results support the existence of RNAa-related mechanism in plant cells, but definitive conclusions can only be drawn after further research.

### 3.3. Plant Gene Activation by Oligodeoxynucleotides

While short single-stranded DNA oligonucleotides (ODNs) are not RNA molecules, they are included in this review because they may produce a functionally similar outcome to RNAa-related processes. ODNs and small RNAs (siRNAs, saRNAs, and miRNAs) are all short nucleic acids similar in chemical structure. These nucleic acids share similar mechanisms leading to activation of gene transcription, e.g., binding to mRNA, DNA promoter, or associated paRNA, and interact with common proteins mediating either transcription activation or mRNA degradation/stalling. Currently, ODN-mediated gene silencing is widely known in mammals and is a powerful approach in medical sciences [92]. ODNs are engineered and applied to various organisms to control gene expression and influence cellular pathways. Furthermore, short ODNs naturally exist in plant cells as degradation intermediates with possible regulatory functions or enter as extracellular DNA, which suggests that these natural ODNs can possess biological functions. Regarding RNAa, accumulating evidence shows that exogenous application of ODNs of 18–25 nt complementary to coding gene sequences [68] or promoter/UTR regulatory regions [69,70] effectively activates target gene expression. It should be noted that the inhibitory effects of ODNs on plant gene expression have also been reported [93].

Considering ODN-mediated gene activation in plants (Table 1), Wojtasik et al. [68] have shown that application of ODNs by vacuum infiltration upregulated the expression of the β-glucanase gene in flax, *Linum usitatissimum*, by altering its cytosine DNA methylation within the coding gene sequence. The ODNs used were designed in antisense orientation to the coding region of the targeted gene. Subsequently, the finding by Dzialo et al. [69] confirmed the capability of exogenously introduced ODNs via vacuum infiltration to temporarily induce or silence chalcone synthase (*CHS*) transcript levels in flax plants depending on the targeted *CHS* region. Importantly, ODNs designed to target the 5′- and 3′-UTRs activated *LuCHS* expression, while ODNs targeting the intron of *LuCHS* suppressed its expression, and those complementary to the *LuCHS* coding region had variable, though primarily suppressive, effects. Dzialo et al. [70] demonstrated that the activating capability of the ODNs was strictly correlated with changing DNA methylation in a sequence-specific manner. The effects persisted for extended periods (>48 h), and these ODN-induced changes in both *LuCHS* gene expression and cytosine methylation could be inherited by flax subsequent generations. Researchers proposed that the ODNs are hybridized either to the homologous region in the complementary mRNA transcript or the DNA gene region after cellular uptake. The ODN hybridization to the mRNA transcript is believed to serve as a molecular signal for RNA-dependent RNA polymerase (RdRP) recruitment and synthesis of the complementary RNA strand. This presumably leads to the formation of dsRNA, which is a key intermediate in RNA-mediated gene regulation.

The current evidence shows that mechanisms of ODN-mediated activation partially overlap with the pathways involved in RNAa (in particular, through the modulation of DNA methylation and chromatin states). However, the precise mechanism of ODN effects on plant gene expression remains unclarified. Taken together, the documented maintenance of acquired gene activation induced by ODNs supports the existence of RNAa in plants as an epigenetic mechanism capable of activating gene expression.

### 3.4. Positive Gene Regulation by miRNA-Mediated Derepression

Plant miRNAs have been documented as important regulators in RNAi-based gene silencing, significantly influencing plant responses to stress [94] and developmental processes [95]. In contrast to the well-established role of miRNAs in gene downregulation, evidence on their potential roles as positive regulators of gene expression in plants is scarce. To the best of our knowledge, evidence for direct positive miRNA roles in the regulation of plant gene expression is lacking, but some studies (Table 1) provide data on indirect positive post-transcriptional gene regulation by protective plant miRNAs [71] or by target mimicry [72]. For example, Couzigou et al. [71] revealed that a mismatched cleavage site in miR171b prevents it from downregulating its target gene, *LOST MERISTEMS 1* (*LOM1*), leading to enhanced *LOM1* expression in alfalfa and tobacco and increased mycorrhization. miR171b is specifically expressed in root cells containing arbuscules and was proposed to have important roles in the establishment of arbuscular mycorrhizal symbiosis, which associates the roots of most land plants with fungi of the phylum Glomeromycota. It has been proven that miR171b protects *LOM1* from inhibition by other more potent members of the miR171 family.

A different study by Franco-Zorrilla et al. [72] demonstrated that the *IPS1* non-coding gene (*Induced by Phosphate Starvation 1*) contains a motif with complementarity to miR399 induced by phosphate deficiency, but pairing is interrupted by a mismatched loop at the putative cleavage site. *IPS1* RNA did not cleave, but sequestered miR399, as a result of which overexpression of *IPS1* has led to increased accumulation of miR399 target *PHO2* mRNA, and at the same time to a decrease in the phosphate content in shoots. The authors coined the term “target mimicry” to describe this mechanism of inhibition of miRNA activity. These results reveal a new mechanism of indirect positive regulation of miRNA-targeted genes via miRNA-mediated derepression, which challenges the traditional concept. It is likely that such miRNAs may exist in other plant miRNA families and that they may be part of a widespread mechanism for regulating gene silencing by miRNAs. Thus, emerging research indicates a more complex form of gene regulation by small RNAs in plants and other organisms than gene silencing.

In animals, pri-miRNAs are processed in the nucleus into shorter pre-miRNA precursors, which are then exported to the cytoplasm where they undergo processing to finally become mature single-stranded miRNAs and exert their biological functions [96]. However, certain animal mature miRNAs contain structural signals that allow their re-entry to the nucleus to perform certain biological functions [97,98]. In plants, unlike in animals, the entire processing of pri-miRNAs into mature miRNAs takes place completely within the nucleus [28,99]. Thus, according to recent studies, mature miRNAs can be located in the nucleus both in plants and animals (Figure 1), where, according to recent animal studies, they participate in transcriptional gene silencing or gene activation [21,22]. This finding considerably expands the landscape of miRNA functions. While direct miRNA-mediated regulation of gene transcription in the nucleus has been confirmed only for animals, the evidence for the presence of mature nuclear miRNAs in plants suggests their involvement in transcriptional gene regulation. Further studies are needed to shed light on the possible nuclear functioning of plant miRNAs.

### 3.5. A Comparative Summary of Experimental Evidence for Plant RNAa-Related Mechanisms

To clarify the strength of experimental support for each proposed plant RNAa-related mechanism, Table 1 summarizes data from representative studies. For each study, we indicate whether changes in transcriptional effect, small RNAs, AGO dependence, locus methylation, functional regulatory elements, and physiological/biochemical effect were experimentally demonstrated (“verified”), inferred from indirect evidence or prior pathway knowledge (“suggested”), not assessed (“not assessed”), or assessed but not detected (“not detected”) in each specific study. As Table 1 illustrates, an increase in gene expression or transcriptional activation was consistently verified across all the studies considered, since this was the defining criterion for their selection. Physiological (whole-plant phenotype) and/or biochemical (metabolite/enzyme-activity) effects were verified in most of the studies. The nucleic acid/DNA methylation-induced gene activation resulted in floral organ identity change, altered callose content, increased mycorrhization, decreased Pi content in shoots, or developmental defects. Also, two studies revealed that the gene activation effects due to DNA methylation have been heritable across plant generations [60,70].

Locus methylation and functional regulatory elements have been verified or suggested in the majority of studies, reflecting that these two features play an important role in current mechanistic understanding of RNAa-related processes in plants. In contrast, no surveyed study has verified a dependence on AGO or other components. In each case, such a dependency is either inferred by analogy to canonical RdDM or miRNA biology, or the data do not support a positive dependency, as in Wojtasik et al. [68]. This represents a significant unresolved gap in the current evidence for plant RNAa-related processes, and this is one of the priorities for future genetic validation (e.g., testing activation phenotypes in ago loss-of-function backgrounds and AGO expression analysis).

## 4. A Comparison of RNA Activation with RNA Interference Processes

RNAa and RNAi are two opposite mechanisms across eukaryotes for regulating gene expression mediated by dsRNAs (i.e., leading to gene activation in RNAa and gene silencing in RNAi), which nevertheless share part of the molecular machinery (Figure 1). Both the RNAa and RNAi processes demonstrate the involvement of the RISC components (e.g., AGO and RHA proteins). However, their distinct kinetic profiles and inherent molecular effects (activation of transcription vs. mRNA degradation) suggest that the RNAa and RNAi phenomena rely on separate signaling mechanisms. Both processes use small dsRNAs of similar size with two nucleotide overhangs at the 3′-end and AGO family proteins; however, RNAi acts primarily in the cytoplasm through RISC against mRNA, while RNAa acts in the nucleus through protein complexes such as RITA against promoters and other regulatory DNA regions (Figure 1). Unlike RNAi, the kinetics of RNAa are characterized by a delayed initiation and a stable effect that persists over several cell divisions, reflecting its epigenetic nature. While siRNAs induce gene silencing in a few hours, the transcriptional response in RNAa is observed only after 1–2 days following saRNA transfection, with transcriptional effects more durable in the latter.

Currently, it is not known under what conditions small RNAs do not provide silencing but induce gene activation. It is possible that the direction of regulation (activation or silencing) in RNA-mediated gene regulation is determined by factors such as the target region (promoter, intron, UTR, or coding region), dsRNA length, DCL processing, AGO loading, RNA stability, and DNA methylation. For example, in plants, DCL3 typically produces 24-nt siRNAs that direct RdDM, DCL4 generates 21-nt siRNAs associated with PTGS (e.g., trans-acting siRNAs), and DCL2 produces 22-nt siRNAs involved in secondary siRNA amplification and mobile silencing signals. DCL1, in contrast, is primarily dedicated to the biogenesis of ~21-nt miRNAs rather than siRNAs, although crosstalk among these pathways has been reported [100]. It is therefore possible to expect that, in certain contexts, 24-nt siRNAs acting through RdDM could lead to transcriptional gene activation (via methylation of a negative cis-element), whereas 21-nt small RNAs are more typically associated with classical silencing. In plants, 24-nt siRNAs are loaded into the AGO4/AGO6 within the RdDM pathway and directed to Pol V transcripts [78,100], while in mammals, AGO2 is the key effector in RNAa [1,38]. It can be assumed that loading into different AGOs in plants can also direct the complex along the path of silencing or activation, but this requires experimental verification.

In contrast to RNAi in plants, which is a mechanistically established pathway with defined core components (e.g., DCL, AGO, RdRP) and abundant experimental validation, RNAa-related processes in plants currently rest on a small and heterogeneous body of evidence. This asymmetry indicates that plant RNAa-related gene activation, as currently defined, is best regarded as an emerging, mechanistically unresolved phenomenon rather than a confirmed regulatory pathway, and that clarifying whether it constitutes a genuine RNA-triggered gene activation mechanism, or is instead a context-dependent reflection of RdDM/chromatin state, should be a priority for future plant molecular biology research.

## 5. Conclusions

Existing research supports the existence of RNA-triggered gene expression activation or RdDM-associated transcriptional derepression at certain plant loci. However, it remains to be determined whether these phenomena constitute a unified pathway analogous to classical animal RNAa, particularly regarding their endogenous triggers, effector complexes, and universality. The literature analysis revealed that the natural occurrence of RNAa in plants is still a prominent unanswered question. Some natural saRNAs or activating miRNAs in plants have not been identified, but existing data on RNAa-related processes and numerous natural antisense transcripts and other non-coding RNAs [101] suggest that additional, uncharacterized RNA-mediated gene activation mechanisms likely exist in plants and are yet to be discovered.

It should be emphasized that in the plant section, the review includes phenomena of different natures, i.e., RdDM-triggered gene activation, exogenous dsRNA-mediated activation, miRNA-mediated derepression, DNA methylation-associated reader complex, and ODN-mediated activation within the scope of the discussion of RNAa. These phenomena are not entirely the same in terms of molecular mechanisms and strength of evidence but could shed light on the natural gene activation processes in plants. On one hand, the available evidence suggests that RdDM-associated transcriptional derepression [60] represents one documented route by which RNA can contribute to gene activation in plants: methylation of specific cytosines in negative cis-elements can block the binding of transcriptional repressors, leading to derepression and activation of the associated gene, with the potential formation of inherited epialleles. However, a mechanistically distinct route has also been described [73,74], in which methylated DNA is directly recognized by reader proteins SUVH1/SUVH3 that recruit DNAJ-domain transcriptional activator proteins, forming complexes with demonstrated transcriptional activation activity at promoter-methylated loci. Thus, DNA methylation in plants can be associated with gene activation both indirectly, through loss of repressor binding, and directly, through recruitment of a positive-acting reader complex. Whether either of these RdDM-associated mechanisms should be considered equivalent to, or merely one manifestation of, the broader plant RNAa phenomenon remains to be clarified.

Further investigation of RNAa-related processes could contribute to understanding of small RNA-regulated cellular processes and gene regulation in plants generally. In addition, the review of current research on gene transcriptional activation in plants shows that further research is needed to understand the effects of exogenous nucleic acids on gene expression.

## Figures and Tables

**Figure 1:**
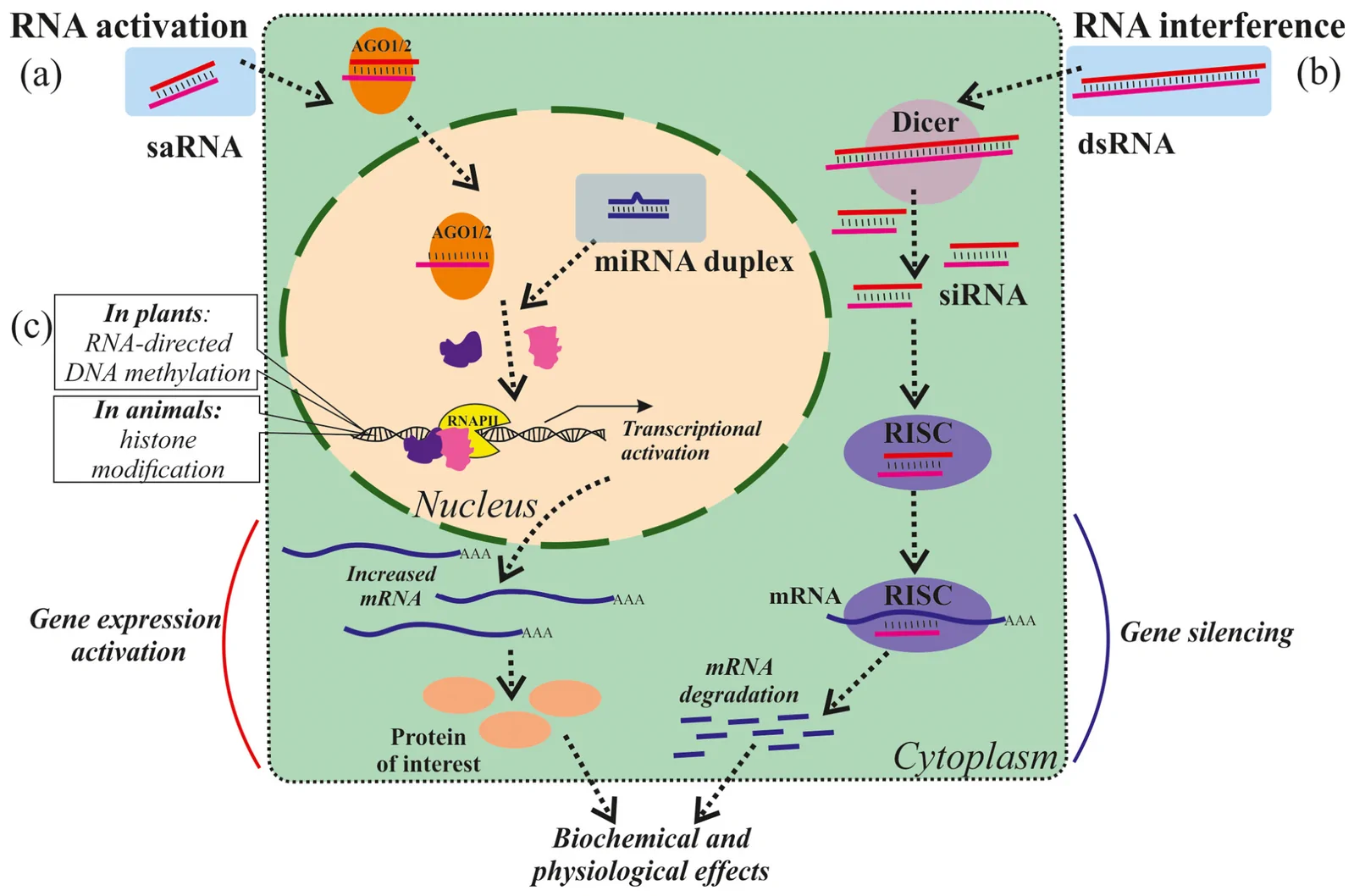
Schematic comparison of the general mechanisms of action for RNA activation (RNAa) and RNA interference (RNAi). (**a**) RNAa occurs in the nucleus, where small RNAs, represented by small activating RNAs (saRNAs) or endogenous activating miRNAs, trigger transcriptional activation. Small RNAs associated with AGO1 or AGO2 bind promoters directly or interact with promoter-associated RNAs. This targeting facilitates the assembly of an activation protein complex, which subsequently interacts with RNA polymerase II (RNAPII) to trigger transcriptional activation. (**b**) In contrast, RNAi occurs primarily in the cytoplasm, where double-stranded RNA precursors are recognized and cleaved by the endonuclease Dicer or DCL to generate siRNAs or miRNAs. Following processing, siRNAs guide the RNA-induced silencing complex (RISC) to cleave complementary mRNA targets. In miRNA-mediated RNAi, RISC is recruited to either induce target mRNA cleavage or repress translation. (**c**) Representative epigenetic features reported in mammalian RNAa and proposed plant RNAa-related processes; the distinction remains provisional because direct mechanistic evidence in plants is limited.

**Table 1:** Existing studies on plant RNAa-related mechanisms: RdDM-triggered gene activation, exogenous dsRNA-mediated activation, indirect positive gene regulation by miRNA-mediated derepression, DNA methylation-associated reader complex, and oligonucleotide-based gene activation.

System	Target	Method	Gene-Expression Effect	Small RNAs	Locus Methylation	Functional Regulatory Elements	AGO Dependence	Mechanism	Physiological/Biochemical Effect	Reference
Petunia *Petunia hybrida* plants	*pMADS3* transcription factor gene (floral homeotic gene)	Plant transformation; transgene-induced, homology-dependent modulation	Verified. Ectopic activation of the *pMADS3* gene.	Suggested. 24-nt siRNAs via the canonical RdDM pathway	Verified. RdDM-induced CG methylation at a single, specific cytosine within pMADS3 intron 2	Suggested. Putative cis-regulatory CpG element in intron 2	Suggested. Canonical AGO4–RdDM link;	RdDM-triggered gene activation, endogenous hairpin-derived siRNAs	Verified. Floral organ identity change; methylation and activation heritable across generations	Shibuya et al. [60]
Tomato *Solanum* *lycopersicum* L. plants	*SlTRY* gene (MYB repressor of anthocyanin biosynthesis)	Foliar spraying of long dsRNAs targeting *SlTRY* intron	Verified. Increased expression of *SlTRY*	Suggested. siRNA involvement; Exogenous synthetic long dsRNA applied	Not assessed.	Suggested. Putative regulatory elements in intron	Suggested. dsRNA-triggered RNAi canonically requires host AGO for siRNA loading	Exogenous dsRNA-mediated activation: long dsRNAs targeting gene regulatory regions	Not detected. Anthocyanin content measured	Suprun et al. 2024 [61]
Flax *Linum usitatissimum* plants	β -glucanase gene	Exogenous application of ODNs targeting the gene coding region by vacuum infiltration	Verified. Upregulated the expression of β -glucanase gene	Suggested. Synthetic ODNs applied, not natural sRNAs; putative siRNA involvement. natural sRNAs	Verified. **G**ene-body CCGG demethylation at exonic/intronic sites; total demethylation	Verified. Gene-body methylation sites, not promoter	Not detected.	ODN-mediated activation by altering cytosine DNA methylation	Verified. Altered callose content.	Wojtasik et al. [68]
Flax *Linum usitatissimum* plants	Chalcone synthase (*CHS*) gene	Exogenous application of ODNs by vacuum infiltration	Verified. Activation of *LuCHS* gene expression	Suggested. Synthetic ODNs, not natural sRNAs; putative siRNA involvement	Verified. Methylation changes at the CCGG sites in the *CHS* regulatory region	Suggested. Putative regulatory elements in 5′-/3′-UTRs of *LuCHS*	Not assessed.	ODN-mediated activation by altering cytosine DNA methylation	Not detected. methylation and activation heritable across generations	Dzialo et al. (2017) [69] Dzialo et al. (2019) [70]
Tobacco and alfalfa *Medicago truncatula*	*LOST MERISTEMS 1* (*LOM1*) gene	Sequence/expression analysis combined with transgenic assays	Verified. *LOM1* enhanced expression	Verified. miR171b	Not assessed.	Verified. Mismatched target/cleavage site in LOM1 mRNA.	Suggested. Canonical AGO1-loaded miRNA action assumed; no ago mutant tested	Indirect positive gene regulation by miRNA-mediated derepression (natural protective miRNAs)	Verified. Increased mycorrhization	Couzigou et al. [71]
*Arabidopsis thaliana *	*PHO2* gene (target miR399), regulated via IPS1	Endogenous IPS1 non-coding RNA, *IPS1* overexpression	Verified. Increased accumulation of *PHO2* mRNA target	Verified. miR399	Not assessed.	Verified. Mismatched complementary site in IPS1 non-coding RNA—acts as a target mimic	Suggested. miR399 is canonically AGO1-loaded; AGO1 not directly manipulated in this study	Indirect positive gene regulation by miRNA-mediated derepression (miRNA sponge/target mimicry)	Verified. Decreased Pi content in shoots	Franco-Zorrilla et al. 2007 [72]
*Arabidopsis thaliana*	Genes proximal to methylated/TE-associated regions	Comparative interactomics (methyl-DNA pulldown/mass spec), genetic mutants, RNA-seq	Verified. Increased expression of genes proximal to methylated/TE-associated regions	Suggested. **L**ink to RdDM-derived siRNAs upstream	Verified. Gene-proximal euchromatic DNA methylation)	Verified. SUVH–DNAJ protein complex that binds non-CG/CG-methylated DNA and acts as a functional regulatory unit	Suggested. **M**ethylation being read is deposited via AGO4-dependent RdDM upstream.	RdDM-linked downstream reader mechanism (SUVH binds methylated DNA and recruits DNAJ1/DNAJ2 to enhance transcriptional activity of the adjacent genes)	Verified. Ectopic overexpression causes developmental defects	Harris et al. (2018) [73]
*Arabidopsis thaliana*	Promoter methylated genes (e.g., *ROS1*)	Chromatin immunoprecipitation combined with sequencing	Verified. Transcriptional gene activation	Suggested. Link to RdDM-derived siRNAs upstream	Verified. Promoter methylation at RdDM-targeted loci)	Verified. SUVH1 binding at methylated promoters.	Suggested. Same reasoning as in Harris et al. [73]	RdDM-linked downstream reader mechanism (Same as in Harris et al. [73].	Verified. Required for normal plant development; protection of promoter methylated genes from silencing	Zhao et al. 2019 [74]

**Note:** Verified—the specific study provides direct experimental data demonstrating this aspect. Suggested—the aspect is inferred from indirect or prior/general pathway evidence, or explicitly described by the original authors as putative, without direct confirmation in that study. Not assessed—the aspect was not assessed in that study. Not detected—the aspect was assessed but not detected in that study.

## Data Availability

The data presented in this study are available within the article and Supplementary Materials.

## Supplementary Materials

The following supporting information can be downloaded at: https://www.mdpi.com/article/10.3390/plants15192956/s1, Table S1: Parameters and effects of RNA activation: comparative data across mammalian, invertebrate, and insect models.

## Abbreviations

AGO	Argonaute protein
Dicer	Dicer-like ribonucleases
DCL	Dicer-like protein
dsRNA	Double-stranded RNA
ODN	Short single-stranded DNA oligonucleotide
paRNA	Promoter-associated RNA
PTGS	Post-transcriptional gene silencing
RISC	RNA-induced silencing complex
RdDM	RNA-directed DNA methylation
RdRP	RNA-dependent RNA polymerase
RNAa	RNA activation
RNAi	RNA interference or gene silencing
RNAPII	RNA polymerase II
RITA	RNA-induced transcriptional activation complex
siRNA	Small interfering RNA
saRNA	Small activating RNA
UTRs	5′ and 3′ Untranslated regions
