# Supplementary material for: RNA Activation in Plants: Current Evidence and Molecular Insights
Source: Plants (Basel). 2026 Sep 28;15(19):2956. doi: 10.3390/plants15192956 (PMC13667248; doi:10.3390/plants15192956)
Supplement: Supplementary file 1 [file plants-15-02956-s001.zip › plants-4488036-supplementary.pdf]

Table S1. Parameters and effects of RNA activation: comparative data across mammalian, invertebrate, and insect models.

System – Target gene	Target region	RNA length (nt)	RNA modification	Magnitude and effect	Citation
**Syntheitc saRNA duplexes**					
Human – E-cadherin, p21, VEGF genes	Promoter, 1–2 kb upstream of TSS	21-nt saRNA duplex, 2-nt 3′ overhangs	Unmodified	Long-lasting, sequence-specific induction (founding RNAa demonstration)	Li et al. 2006 [1]
Human – Progesterone receptor PR gene	Promoter	19–21-nt agRNA duplex	Unmodified	Sequence-specific transcriptional induction	Janowski et al. 2007 [13]
Human/mouse – interleukin-2 (IL-2) gene	TATA-box-centered promoter region	19-23-nt promoter-targeting activating siRNA duplex	2′-O-Methyl modification at the 3′ terminus of the antisense strand (explicitly optimized)	Potently enhanced the expression of IL-2 gene in human and mouse primary CD4+ T cells with a long-time effect	Fan et al. 2014 [45]
Rat – myogenic regulatory factor MyoD	~1 kb promoter scan	21-nt saRNA duplex	Unmodified	MyoD Induced expression of at both the mRNA and protein levels; inhibited cell proliferation of adipose-derived stem cells (ADSCs)	Wang et al. 2015 [46]
Human – E-cadherin (CDH1) tumor suppressor gene	Promoter, −1000 to −100 bp; algorithmic scan, 88 top candidates screened	19-nt saRNA duplex, 2-nt 3′ overhangs	Unmodified	Induction of CDH1 expression in human prostate cancer cell line	Wang et al. 2015 [47]
Human – dihydro-pyrimidinase-like 3 gene (DPYSL3), a cancer suppressor	Promoter	19-21-nt saRNA duplex	Unmodified	Enhanced DPYSL3v2 expression; reduced cell mobility in multiple prostate cancer cell lines	Li et al. 2016 [48]
Human – the progesterone receptor (PR) gene	~ 2 kb of the PR promoter and sites around the TSS	21-nt saRNA duplex, 2-nt 3′ overhangs	Unmodified	Activation of PR gene; saRNAs bound to promoter in a seed-region-dependent manner	Meng et al. 2016 [38]
Human – transcription factor CCATT/enhancer binding protein alpha (CEBPA)	Promoter; bioinformatic "nucleotide walk" across gene	19–21-nt saRNA duplex length	2′-O-Methyl modification specifically to prevent activation of innate immunity	2.5-fold CEBPA mRNA increase in hepatocellular carcinoma (HCC) cell lines; first-in-human trial responses	Voutila et al.2017 [49]
Human – CEBPA gene	Coding sequence	21-nt saRNA duplex	Chemically modified (clinical-grade); combined with DNA-demethylation readout	Elicits transcriptional activation of CEBPA gene through promotor demethylation	Gaggi et al. 2025 [50]
Human – vitamin D3 up-regulated protein 1 (VDUP1) gene	Promoter	21-nt saRNA duplex	Unmodified	Up-regulated expression of VDUP1 at both mRNA and protein levels; cell-cycle arrest and reduced proliferation in lung cancer cells	Park et al. 2022 [51]
**Synthetic and biogenic endogenous miRNA duplexes**					

Human – E-cadherin, CSDC2 genes	Promoter; natural miRNA, site at −645 relative to TSS	~22-nt mature miRNA (miR-373) / precursor hairpin	Unmodified (native miRNA)	Robust induction of both genes	Place et al.2008 [16]
Mouse – Ccnb1	Promoter elements	~22-nt mature miRNAs (miR-744, miR-1186, miR-466d-3p)	Unmodified	Ccnb1 induction across cell lines	Huang et al. 2012 [52]
C. elegans – germline genes	Mature mRNA body — not promoter/intron	22 nt (endogenous "22G-RNAs")	Unmodified (endogenous, biogenic)	Maintains active chromatin state, not acute fold-change	Seth et al. 2013 [53]
Human – CDH1 gene	Same promoter site as above (Place et al.2008)	~22-nt mature miRNA (miR-373)	Unmodified	Significantly suppresses the proliferation and metastasis capacity of human bladder cancer cells mainly by stimulating CDH1 expression	Zhang et al. 2017 [54]
Human – FBP1, FANCC	Enhancer (H3K27ac/H3K4me1-marked regulatory element, not promoter)	~22-nt mature miRNA (miR-24-1)	Unmodified (native miRNA mimic)	Ectopic miR-24-1 expression up-regulated ~1074 genes genome-wide (per follow-up synthesis)	Xiao et al. 2017 [15]
Human – genome-wide enhancer network, validation of Xiao 2017	Enhancer, core-sequence-dependent	~22-nt (miR-24-1)	Unmodified	Confirms enhancer (not promoter) as the operative target region	Liang et al.2017 [24]
Human – IL24, IL32	Promoter	~22-nt mature miRNA (miR-205)	Unmodified	Tumor-suppressor gene induction	Tan et al. 2019 [55]
Insect *Aedes aegypti* (Aag2 cells)	OpIE2 promoter (exogenous GFP) + endogenous immune gene promoters	Long dsRNA (hundreds of bp) and short saRNA (~21 nt)	Unmodified	Activation of both exogenous and endogenous targets	De Hayr et al. 2020 [56]
Tick *(Haemaphysalis longicornis)* - HlemCHT endochitinase-like gene	Promoter + 3′-UTR AU-rich elements	saRNA/dsRNA, variable length	Unmodified	Increased *HlemCHT* gene expression	Kwofie et al. 2023 [57]
Human – CYP19A1	Promoter	~22-nt mature miRNA (miR-339)	Unmodified	Increased CYP19A1 transcription, increased estrogen release	Wang et al. 2023 [58]
Human – MMP1	Enhancer (H3K27ac-marked)	~22-nt mature miRNA (miR-1246)	Unmodified	~2-fold increase in migratory capacity attributable to miR-1246	Yang et al., 2024 [59]

Note: TSS – transcription start site; saRNA – small activating RNA; miRNA – microRNA; agRNA – antigene RNA
